# Facile Preparation of Carbon Nanotubes/Cellulose Nanofibrils/Manganese Dioxide Nanowires Electrode for Improved Solid-Sate Supercapacitor Performances

**DOI:** 10.3390/polym15183758

**Published:** 2023-09-14

**Authors:** Siew Xian Chin, Kam Sheng Lau, Riski Titian Ginting, Sin Tee Tan, Poi Sim Khiew, Chin Hua Chia, Chatchawal Wongchoosuk

**Affiliations:** 1 Department of Physics, Faculty of Science, Kasetsart University, Chatuchak, Bangkok 10900, Thailand; chinsiewxian@ukm.edu.my; 2 ASASIpintar Program, Pusat GENIUS@Pintar Negara, Universiti Kebangsaan Malaysia, Bangi 43600, Selangor, Malaysia; 3 Materials Science Program, Faculty of Science and Technology, Universiti Kebangsaan Malaysia, Bangi 43600, Selangor, Malaysia; kamshenglau@gmail.com; 4 Department of Electrical Engineering, Universitas Prima Indonesia, Medan 20118, North Sumatra, Indonesia; 5 Nanomaterials for Renewable Energy (NRE) Laboratory, Medan 20133, North Sumatra, Indonesia; 6 Department of Physics, Faculty of Science, Universiti Putra Malaysia, Serdang 43400, Selangor, Malaysia; tansintee@upm.edu.my; 7 Center of Nanotechnology and Advanced Materials, Faculty of Engineering, University of Nottingham Malaysia Campus, Jalan Broga, Semenyih 43500, Selangor, Malaysia; poisim.khiew@nottingham.edu.my

**Keywords:** cellulose nanofibrils, supercapacitor, MnO_2_ nanowires, paper electrode

## Abstract

Wearable energy storage devices require high mechanical stability and high-capacitance flexible electrodes. In this study, we design a flexible supercapacitor electrode consisting of 1-dimensional carbon nanotubes (CNT), cellulose nanofibrils (CNF), and manganese dioxide nanowires (MnO_2_ NWs). The flexible and conductive CNT/CNF-MnO_2_ NWs suspension was first prepared via ultrasonic dispersion approach, followed by vacuum filtration and hot press to form the composite paper electrode. The morphological studies show entanglement between CNT and CNF, which supports the mechanical properties of the composite. The CNT/CNF-MnO_2_ NWs electrode exhibits lower resistance when subjected to various bending angles (−120–+120°) compared to the CNT/CNF electrode. In addition, the solid-state supercapacitor also shows a high energy density of 38 μWh cm^−2^ and capacitance retention of 83.2% after 5000 cycles.

## 1. Introduction

Energy storage with efficient conversion has emerged as a growing research area to meet the demands of modern technology and to address the issues related to sustainable energy [1]. Energy storage and energy harvesting are two different topics with widespread applications, including mechanical to electrical energy and electrical to electrochemical energy conversion and on the same platform [2]. For the past few years, supercapacitors have attracted a great deal of research interest in the field of energy storage owing to their low cost, long cycle life, higher power, and energy density [3]. Supercapacitors (SC) are promising technology for next-generation flexible storage devices owing to their high-power density, rapid charging/discharging, and longer cycle life than dielectric capacitors and batteries [4,5].

In general, supercapacitors can be divided into two categories, namely electrical double-layer capacitors (EDLCs) and pseudocapacitors depending on the active materials and charge storage mechanism [6]. EDLCs, for example, use carbon as the electrode material and do not undergo redox reactions during the charge–discharge process, but instead store and release energy through the rapid adsorption/desorption process of electrolyte ions on the surface of the electrode material in contact with the electrolyte [7,8]. As a result, this type of supercapacitor has a high-power density and a very quick charge and discharge rate, allowing it to complete the full charge and discharge operation in a relatively short period. The surface area of the electrode in contact with the electrolyte and adsorbed ions is an important factor that defines the energy storage capacity of such a supercapacitor, while the conductivity and particular structure of the electrode material are secondary factors [9]. The primary drawback of EDLCs is their lower energy density, hence the development of pseudocapacitors-based supercapacitors, which enable the charge and discharge process on the electrode surface via a fast and reversible redox reaction. This type of supercapacitor has greater energy storage capability due to the redox reaction during the charge and discharge process and mostly employs metal oxides and conductive polymers as electrode materials, and the surface chemicals of these materials allow the redox reactions to occur. It has a high energy density, often three times that of EDLCs [10]. However, the pseudocapacitors have redox processes that require more time for the charge and discharge time than those of EDLCs, which lowers their power density. At the same time, the electrode materials of pseudocapacitors supercapacitors are prone to active material separation and three-dimensional shape degradation after various cycles of charge–discharge processes [11].

Flexible supercapacitor electrodes have been designed and made for flexible energy storage devices. In general, flexibility refers to the capacity of materials or devices to distort. Bending, stretching, or softness can all be used to illustrate flexibility. The link between bending ability, stretchability, and softness is related but not identical. A soft gadget, for example, is normally bendable, but not vice versa. Typically, the capacity of materials to bend is used to demonstrate flexibility [12]. The use of various active materials, such as carbon nanomaterials (graphene and carbon nanotubes (CNT)), conducting polymers (polyaniline and polypyrrole), and transition metal oxides (MnO_2_, MoO_3_, etc.) allows the formation of these flexible materials due to the material’s unique structures [13,14,15,16]. However, most of these active materials are unable to form a free-standing film that is mechanically stable. As a result, several attempts have been made to construct flexible electrodes supported by flexible substrates, namely paper, fabric, yarn, and different polymeric films serving as these flexible substrates [17]. Although this does not add to the capacitance value, these substrates contribute significantly to the weight of the device, which results in a low specific capacitance depending on the weight of the entire device [18,19]. Therefore, it is essential to design freestanding electrodes with high specific capacitance, robust mechanical flexibility, and outstanding cycle stability for the fabrication of high-performance flexible supercapacitors [20]. Furthermore, the aerogel-structured composite is a 3D solid network prepared by the sol–gel method [21]. This type of aerogel structure is considered a good substrate material in many fields due to the ultra-high specific surface area and excellent mechanical properties, which can be loaded with capacitor active electrode materials, which have good energy storage properties [22].

One-dimensional (1D) material has been of great interest to researchers to explore applications due to its robust flexibility by having a high aspect ratio geometry [23,24]. Carbon nanotubes (CNTs) made up of sp^2^ hybridized carbons possess high surface area (1300 m^2^·g^−1^), high conductivity, and chemical stability, which is a suitable material for energy storage applications based on the electrical double-layer principle [25,26]. However, CNT tends to form a rigid film when it is used as electrode material, hence the incorporation of 1D cellulose nanofibrils (CNF) will help to improve the flexibility and mechanical strength of the composite film [27,28]. Cellulose is a common linear biopolymer composed of glucose units that, in nature, form highly organised, long, and thin nanostructures. CNFs are well-known for their low thermal expansion coefficient and high rigidity (elastic modulus of 138 GPa) [29]. They generally have a width of 4 nm and a length of 500–2000 nm when recovered from delignified wood fibres, depending on the raw material used and how it was treated [30]. Because CNF are closely packed in the lamellae of the fibre wall, their separation necessitates expensive and high-energy mechanical treatments. The inclusion of charged moieties in the cellulose backbone, on the other hand, can dramatically lower the cost of the extraction process. These charges cause electrostatic repulsion between neighbouring CNF, thereby counteracting the strong inter-fibrillar interactions that keep the fibre wall together and allow for the rapid extraction of individualised CNF. The production of a CNF-based electrode with various conductive materials has been performed by various researchers. Zu et al. (2022) prepared a CNF and CNT porous hybrid foam using physical blending via an ultrasonic homogenizer and then solution casting into a mould, which allowed the mixing solution subjected to freeze drying to obtain the hybrid foam sample [31]. Furthermore, conductive paper materials that were produced by Chen et al. 2020 using CNF and silver nanowires, which were mixed in aqueous dispersions and filtered to produce the film sample, exhibited a low electrical conductivity of 1.7 × 10^−8^ S/cm [32]. Li et al. (2020) prepared a flexible heterogeneous multilayered film consisting of CNF and graphene nanosheets via the alternating vacuum filtration process. The electrical conductivity of the film can increase approximately 7–11 times when the number of conductive layers is increased; however, the increasing ratio of graphene nanosheet to CNF content will inhibit good electrical conductivity due to the imperfect creation of layered conductive path in conductive layers [33]. Xu et al. (2019) reported a cellulose nanofiber aerogel composite prepared via the supramolecular self-assembly approach using polyaniline suspension mixing. The lightweight conductive supramolecular aerogel with hierarchically porous 3D structures demonstrated a high conductivity of 0.372 mS/cm and a larger area-normalized capacitance of 59.26 mF/cm^2^. The flexible supercapacitor device can achieve a high normalised capacitance of 291.01 F/g using a flexible solid electrolyte consisting of a polyvinyl alcohol (PVA) solid matrix and a sulfuric acid electrolyte. The supramolecular cellulose nanofiber aerogel produced exhibited fast charge–discharge performance and excellent capacitance retention, which is better than other 3D chemically cross-linked nanocellulose aerogels [34].

To further improve the energy density without sacrificing the power density of supercapacitor devices, combining CNT with other pseudocapacitive metal oxide materials would be a promising approach to produce a better-performing supercapacitor. Manganese dioxide nanowires (MnO_2_ NWs) attracted researchers to use them as an active material in supercapacitors due to their high theoretical specific capacitance of 1400 F g^−1^ and natural abundance [35,36]. Lv et al. (2018) designed an editable and flexible supercapacitor electrode based on ultralong MnO_2_ NWs and a CNT composite prepared via solution mixing and vacuum filtration. This supercapacitor device, which can achieve a specific capacitance of 227.2 mF cm^−2^ and maintain ~98% performance after 10,000 stretch cycles, used polyvinyl alcohol/lithium chloride (LiCl) as an aqueous gel polymer electrolyte [37]. Sui et al. (2020) developed nitrogen-doped carbon nanowires with MnO_2_ via electrodeposition of MnO_2_ on the carbon dioxide-activated polypyrrole nanowires. The asymmetric supercapacitor, which achieves an energy density of 23.7 Wh kg^−1^ at 2000 W kg^−1^, used a sodium sulphate electrolyte solution [38]. Sajjad et al. (2021) fabricated a 2 V aqueous phosphine-based porous organic polymer/reduced graphene oxide/α-MnO_2_ asymmetric supercapacitor with cycling stability of 96% retention (10,000 cycles) and a high specific energy of 39 Wh kg^−1^ [39]. Jayachandran et al. (2021) studied the effect of various aqueous electrolytes on the electrochemical of α-MnO_2_ nanorods, which achieved specific capacitance of 570 F g^−1^ at 1 A g^−1^ current density in the mixture electrolyte (1 M sodium sulphate (Na_2_SO_4_) and 0.5 M potassium hydroxide (KOH)) and maintained capacitance retention of ~80% after 10,000 cycles [40]. He et al. (2022) reported a hybrid cathode material consisting of 2D Ti_3_C_2_T_x_ nanosheets and 1D MnO_2_ nanobelts prepared via a colloidal solution filtering approach to generate an alternating MnO_2_/Ti_3_C_2_T_x_ stacked structure as a flexible electrode for a supercapacitor [41]. The hybrid cathode is more electrochemically stable toward anodic oxidation than pure Ti_3_C_2_T_x_, which has a high gravimetric capacitance of 315 F g^−1^ at 10 mV s^−1^ and a strong rate capability of 166 F g^−1^ at 100 mV·s^−1^. Carbon fibre/polypyrrole fibre prepared by electrochemical deposition of polypyrrole on the surface of carbon fibre was used as the negative electrode and a CNT/MnO_2_ film prepared by electrochemical deposition of manganese(II) acetate on the surface of the CNT electrode was used as the positive electrode to create a flexible supercapacitor. The device had a maximum areal capacitance of 66.27 mF·cm^−2^ and an areal energy density of 23.56 Wh·cm^−2^, which can maintain capacitance retention of 83% after 5000 cyclic stability tests [42]. Zhao et al. (2022) reported the first liquid–liquid interface deposition with electrostatic self-assembly of a MnO_2_@rGO core-shell nanosphere as material for flexible supercapacitor applications. The built symmetric micro-supercapacitor had a high areal energy density of 1.01 Wh·cm^−2^, with excellent cycling stability and a capacity retention rate of nearly 100% after 2000 bending cycles [43]. 

In this study, a flexible CNT/CNF composite paper was formed via the ultrasonic dispersion approach and combined with hydrothermally grown MnO_2_ NWs as the supercapacitor electrode. The formation of the electrode was achieved using vacuum filtration to obtain the composite film, which was then subjected to freeze drying to remove the excessive water while maintaining the porosity of the electrode sample. Lastly, the composite was hot pressed to form the electrode. The combination of conductive CNT and fibrous CNF can improve the electrical and mechanical properties of the composite film, which allows it to be flexible and simultaneously improves the energy density.

## 2. Materials and Methods

### 2.1. Materials

Kenaf core powders (60–80 mesh) were obtained from the Malaysian Agricultural Research and Development Institute (MARDI). A single-walled carbon nanotube (CNT) with a length-to-diameter ratio of 10^3^–10^4^ was purchased from Tuball™ (Leudelange, Luxembourg). Sodium chlorite (NaClO_2_, 80%) and polyvinyl alcohol (PVA, M_w_ = 86,000 g/mol) were purchased from Acros Organics (Geel, Belgium). Acetic acid (96%), nitric acid (HNO_3_, 65%), acetone (>99.8%), ammonium fluoride (NH_4_F, ≥98%), phosphoric acid (H_3_PO_4_, 85%), sodium chloride (NaCl, ≥99.5%), and sodium sulfate (Na_2_SO_4_, >99%) were purchased from Merck Millipore (Burlington, MA, USA). Potassium permanganate (KMnO_4_, >99%) was purchased from Fisher Scientific (Waltham, MA, USA).

### 2.2. Methodology

#### 2.2.1. Preparation of CNF Dispersion

Kenaf core powder was used to obtain the CNF, which was reported in our previous work [44]. Briefly, kenaf core powder had undergone a delignification process using 1.875 g/g of NaClO_2_ and 1.25 g/g of acetic acid six times. After that, the kenaf core powder was filtered and washed with deionised water and acetone until it reached neutral pH. A mixture solution containing 0.1 wt% of delignified kenaf core powder and 0.1 mM NaCl was prepared for defibrillation to obtain CNF. Then, the defibrillation process was performed using a high-shear laboratory mixer (Silverson, L5M-A, East Longmeadow, MA, USA) at 10,000 rpm for 16 h to produce CNF.

#### 2.2.2. Preparation of CNT Dispersion

First, 0.1 wt% of CNT was dispersed in a 20% HNO_3_ solution using an ultrasonication probe for 30 min. Then, the solution was refluxed at 60 °C for 6 h to remove impurities in the CNT. Subsequently, the CNT solution was filtered and washed with deionised water until a neutral pH was obtained, followed by being oven-dried at 60 °C for 12 h.

#### 2.2.3. Synthesis of MnO_2_ NWs

First, 0.5 mol of KMnO_4_ and 0.5 mol of NH_4_F were dissolved in deionised water and stirred for 30 min. Then, the solution was transferred into a stainless-steel Teflon autoclave. The hydrothermal process was performed at 180 °C for 3 h. Then, the product was washed with deionised water three times via centrifugation at 8000 rpm for 15 min. After that, the MnO_2_ NWs were oven-dried at 60 °C for 12 h.

#### 2.2.4. Fabrication of CNT/CNF Electrode and CNT/CNF-MnO_2_ NWs Electrode

First, 0.07 wt% of CNF and 0.1 wt% of CNT were mixed in a 1:1 weight ratio and then subjected to an ultrasonic probe for 30 min to obtain a homogenous solution. Then, the dispersed solution was filtered using a membrane filter with a diameter of 47 mm to obtain a CNT/CNF film. The film was then freeze-dried for 24 h at −105 °C until all the ice crystals were removed from the film. Then, the freeze-dried film was hot pressed for 10 min at 120 °C. To fabricate the CNT/CNF-MnO_2_ NWs electrode, the procedure was repeated with the addition of a 0.5 ratio of the as-synthesised MnO_2_ NWs.

### 2.3. Characterisation

Thin-film X-ray diffraction (XRD) was performed using an XRD diffractometer (Bruker, AXS D8 Advance, Baden, Switzerland) scanning from 2θ = 5–80° equipped with an X-ray source of Cu K_α_ radiation = 1.5418 Å. The Raman spectra of the samples were obtained using Raman micro-spectroscopy (Technospex, uRaman-Ci, Singapore) equipped with a 532 nm laser and an optical microscope (Nikon Eclipse Ci L, Tokyo, Japan) with exposure power and exposure time of 1 mW and 5 s, respectively. The IR spectra were obtained using a Fourier Transform Infrared (FTIR) spectrometer (Bruker, Alpha, Ettlingen, Germany). The morphologies of the samples were measured using a field emission scanning electron microscope (FESEM, Schottky SU5000, Tokyo, Japan) with an electron high tension of 3 kV. The specific surface area of the electrode was analysed using Brunauer, Emmett, and Teller (BET) analysis (Micromeritics, ASAP 2010, Norcross, GA, USA), with degassing at 353 K for 90 min and then analysed at 99 K. The resistance measurement of the electrode was performed using the setup in a previous report [45]. The samples were placed onto a sample holder, which was subjected to various bending angles from +120 to −120°. The resistance of the sample was measured from the furthest side using a digital multimetre (VC97, VICTOR, Xi’an, China).

An asymmetrical supercapacitor device was fabricated using a CNT/CNF electrode as anode and CNT/CNF-MnO_2_ NWs electrode as the cathode, and a gel polymer electrolyte consisting of 1 M LiCl in PVA solution. The dimension of the electrode was 2 × 1 cm (L × W) with a mass of 9–11 mg. The thickness of the electrode is 0.07–0.08 mm. The cyclic voltammetry (CV) and galvanostatic charge–discharge (GCD) were analysed with a potential window of 0–0.8 V. Electrochemical Impedance Spectroscopy (EIS) analyses were conducted at open circuit potential from a 10,000–0.01 Hz frequency range and an amplitude of 5 mV.

## 3. Results and Discussion

**Figure 1:**
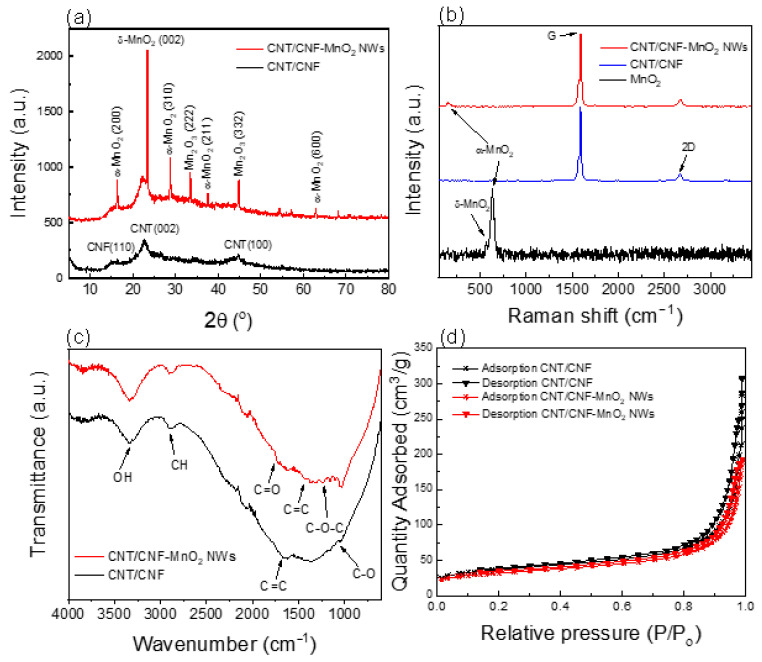
(**a**) XRD diffractogram, (**b**) Raman spectra and (**c**) FTIR spectra of CNT/CNF and CNT/CNF-MnO_2_ NWs composites, and (**d**) Nitrogen adsorption–desorption isotherms of the composites measured at 77 K.

The XRD diffraction peaks for CNT/CNF and CNT/CNF-MnO_2_ NWs composites are shown in Figure 1a. The board peak at 16.3° corresponds to the (110) plane of the CNF, whereas for CNT, the peaks centred at 22.5° and 44.7° correlated to the (002) and (100) plane, respectively. For the CNT/CNF-MnO_2_ NWs electrode, the diffraction peak at 16.3°, 28.7°, 37.6°, and 62.8° represent the tetragonal phase of α-MnO_2_ (JCPDS No. 44-0141) and the peak at 23.4° is due to the presence of δ-MnO_2_ (JCPDS No. 80-1098). This result is consistent with a previous report [46]. Additionally, the observed peaks at 33.4° and 44.8° correspond to Mn_2_O_3_ (JCPDS No. 24-0508).

The Raman spectra (Figure 1b) for MnO_2_ NWs show a peak at 631 cm^−1^, which corresponds to the A_g_ mode of α-MnO_2_. It also shows a shoulder peak around 560 cm^−1^, which corresponds to the A_1g_ mode of δ-MnO_2_ [47]. The presence of α and δ phase MnO_2_ further confirms the findings of the XRD analysis. For the CNT/CNF composite, it shows the typical peaks for carbon nanomaterials. The G band at 1588 cm^−1^ corresponds to the E_2g_ mode vibration of graphitic bonding. There is a small shoulder peak at 1569 cm^−1^, which arose from the interaction between the different CNT shells [48,49]. The 2D band at 2670 cm^−1^ was attributed to the multiple layers of CNT stacking. Moreover, the presence of MnO_2_ NWs in the composite does disturb the order of the graphitic structure in the CNT. Moreover, the peak at 155 cm^−1^ can be ascribed to the dominant external mode from the translational motion of MnO_6_ units in α-MnO_2_ [50].

Figure 1c shows the FTIR spectrum of the composite. The broad peak at around 3000–3500 cm^−1^ corresponding to OH groups’ presence in the CNF could be responsible for the formation of intermolecular hydrogen bonding between CNF. There is no peak shift (3339 cm^−1^) with the incorporation of MnO_2_ NWs, indicating that it does not change the hydrogen bond vibration. The peaks at around 2916, 1241, and 1053 cm^−1^ can be ascribed to the CH, C-OC, and C-OH groups, respectively, due to the presence of glucopyranose monomers in CNF [51]. Interestingly, the CNT/CNF-MnO_2_ NWs showed a visible peak compared to CNT/CNF due to the presence of MnO_2_ decreasing the hydrophilicity of the sample [52], which reduced the absorbed water on the CNF molecules. Furthermore, the peaks around 1666 and 1428 cm^−1^ are attributed to the C=C group in the backbone structure of MWCNT. The peak at around 1730 cm^−1^ corresponds to C=O groups due to the oxidation of CNT in nitric acid solution.

**Figure 2:**
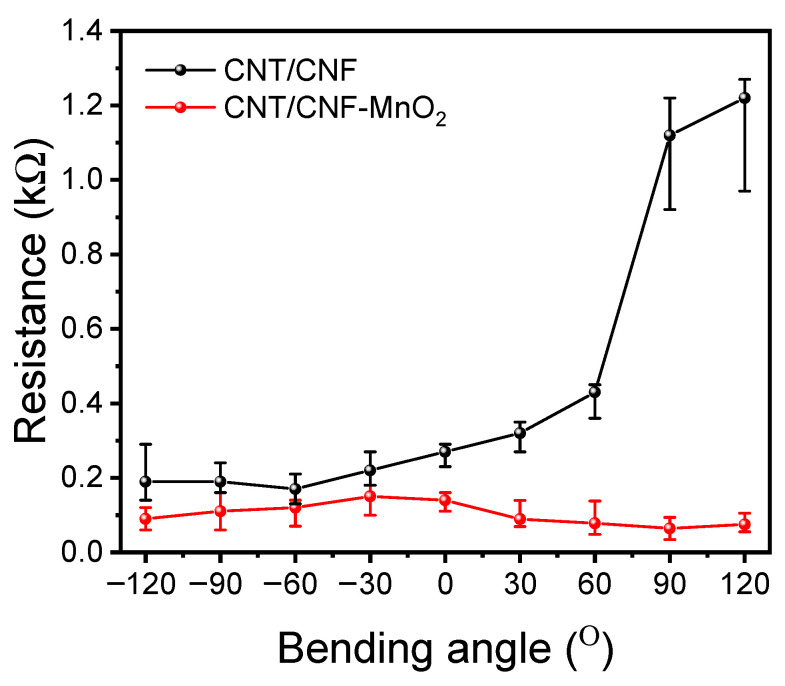
Resistance measurements of CNT/CNF and CNT/CNF-MnO_2_ NWs electrodes as function of bending angle.

Table 1 shows the BET analysis results for the CNT/CNF and CNT/CNF-MnO_2_, which describes the changes in the specific surface area of the electrode with the presence of MnO_2_ NWs. The decreases in BET surface area, pore volume, and pore size are due to the presence of MnO_2_, which could disrupt the arrangement in the CNT/CNF matrix. Interestingly, the resistance of the CNT/CNF-MnO_2_ NWs composite had a lower value compared with the CNT/CNF electrode due to the more compact structural arrangement of CNT in the electrode matrix, which correlates with the BET analysis. Furthermore, the resistance of the electrodes at various bending angles (Figure 2) was measured and the results show that the CNT/CNF MnO_2_ NWs electrode was more stable and robust and maintained the resistance when applied at different bending angles compared to the CNT/CNF electrode. However, for the CNT/CNF electrode, the resistance values at higher bending degrees are asymmetric, suggesting the breakdown of the electrode structure due to multiple consecutive bending from −120° to +120°. 

**Figure 3:**
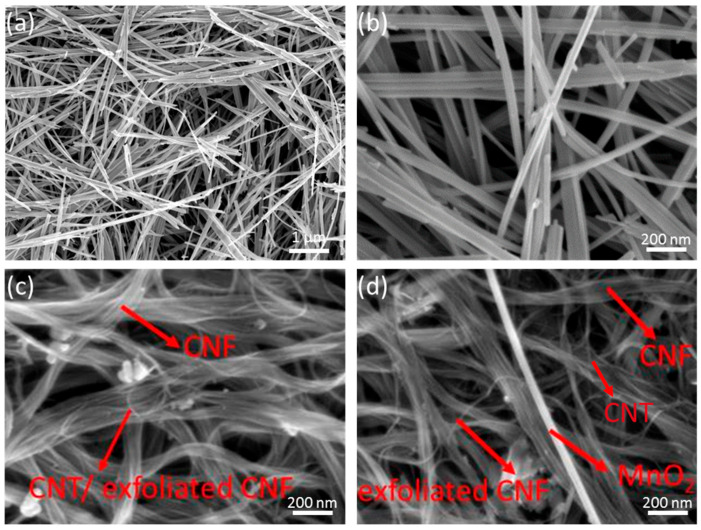
FESEM images of MnO_2_ NWs at lower (**a**) and higher (**b**) magnification, (**c**) CNT/CNF electrode, and (**d**) CNT/CNF-MnO_2_ NWs electrode.

Figure 3a,b show the FESEM images of as-synthesised MnO_2_ NWs_,_ with an average diameter of 40 ± 5 nm and length of ~30 µm, thus an aspect ratio of 750 was obtained. Furthermore, the FESEM image of the CNT/CNF electrode (Figure 3c) indicates the CNT is distributed uniformly parallel to the exfoliated CNF, while the CNT/CNF-MnO_2_ NWs also show a diameter of ~40 nm for MnO_2_ NWs, indicating the ultrasonic probe dispersion process does not introduce structural changes to the MnO_2_ NWs.

**Figure 4:**
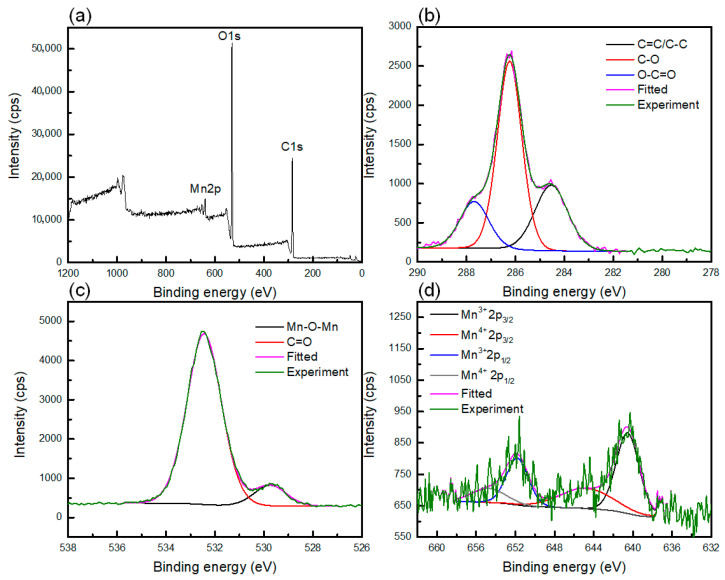
(**a**) Survey XPS spectra of CNT/CNF-MnO_2_ NWs electrode and narrow scan for deconvoluted (**b**) C1s, (**c**) O1s, and (**d**) Mn2p XPS spectra.

The surface chemical composition of the CNT/CNF-MnO_2_ NWs electrode was further analysed by XPS to investigate the oxidation states of MnO_2_ as shown in Figure 4. From the narrow scan of C1s, the deconvoluted peaks at 284.54 eV correspond to the C-C and C=C bonds’ presence in the CNT and CNF. Moreover, the deconvoluted peak at 286.24 eV correlated to the C-O bond arising from ether in the monomer unit of cellulose and glycosidic linkage of the polymer backbone chain of the CNF. The deconvoluted peak at 287.68 eV was ascribed to the carboxylic acid group’s presence in the oxidized CNT. Furthermore, the O1s spectra show two peaks at 532.44 and 529.74 eV, which are attributed to the Mn-O-Mn bond from the MnO_2_ structure and the C=O bond arising from the cellulosic structure of CNF, respectively. Moreover, the Mn2p spectra show that the deconvoluted peaks at 642.38, 646.33, 653.67, and 654.43 eV are ascribed to Mn^3+^2p_3/2_, Mn^4+^2p_3/2_, Mn^3+^2p_1/2_, and Mn^4+^2p_1/2_, respectively. Furthermore, the spin-energy separation calculated from both Mn2p_3/2_ and Mn2p_1/2_ peaks is 11.5 eV, which implied that the Mn^4+^ ion was dominant in CNT/CNF, hence it is in good agreement with a previous study [53].

**Figure 5:**
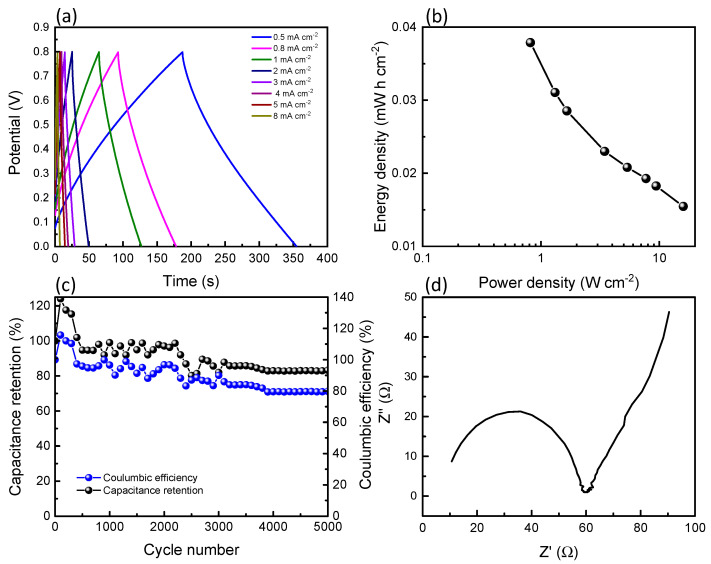
(**a**) GCD measurements at various charge–discharge rates and its (**b**) Ragone plot, (**c**) cyclic stability measured at 2 mA·cm^−2^ for 5000 cycles, and (**d**) EIS plot for asymmetrical device CNT/CNF|PVA-LiCl|CNT/CNF-MnO_2_ NWs.

The electrodes were fabricated into an asymmetrical supercapacitor device to determine the performance and the results are shown in Figure 5. The GCD measurement also shows a symmetrical triangular shape, which is the same as the CNT/CNF-MnO_2_ NWs electrode analysis, but the charge–discharge time decreases due to lower ionic mobility and accessibility in the gel electrolyte compared to the aqueous electrolyte system. The GCD results are displayed in a Ragone plot, and this asymmetrical supercapacitor device can obtain a power density and energy density of 0.81 W cm^−2^ and 0.038 mWh cm^−2^, respectively. Furthermore, it was tested for its cyclic stability for 5000 cycles at 2 mA cm^−2^ and maintained good capacitance retention at 83.2% and a coulombic efficiency of 79.8%, indicating a successful supercapacitor device had been produced. Moreover, the impedance spectrum was fitted with an electrical equivalent circuit showing the solution resistance (R_s_) and charge-transfer resistance (R_ct_) of 10.7 and 21.3 Ω, respectively. Comparison on the results of the solid-state supercapacitor device performance with previous reported studies is tabulated in Table 2.

## 4. Conclusions

In this study, a CNT/CNF electrode was fabricated using an ultrasonic probe dispersion method as a free-standing flexible electrode and α-MnO_2_ NWs were incorporated into the CNT/CNF electrode matrix, which was used as a supercapacitor electrode device. The structural analysis of the MnO_2_ shows the presence of a crystal phase of the MnO_2_, which is beneficial in the electrochemical capacitor properties. The characterisation shows that CNT/CNF-MnO_2_ NWs electrodes have individual nanowires attached to the surface of the CNT/CNF matrix, which allows the maximum exposed surface area of MnO_2_ NWs, which can maintain good electrical properties upon various bending angles. The supercapacitor device can obtain a power density and energy density of 0.81 W cm^−2^ and 0.038 mWh cm^−2^, respectively, and can maintain good capacitance retention at 83.2% after 5000 cycles.

## Figures and Tables

**Table 1:** Nitrogen absorption properties of CNT/CNF and CNT/CNF-MnO_2_ electrodes.

Sample	BET Surface Area (m^2^·g^−1^)	Pore Volume (cm^3^·g^−1^)	Pore Size (Å)
CNT/CNF electrode	129.5	0.331	102.2
CNT/CNF-MnO_2_ NWs electrode	113.0	0.282	100.0

**Table 2:** Comparison of solid-state supercapacitor device performances.

No.	Electrodes	Electrolyte Used	Current Density (mA·cm^−2^)	Areal Capacitance (mF·cm^−2^)	Scan Rate (mVs^−1^)	Current Density (A·g^−1^)	Specific Capacitance (F·g^−1^)	Ref.
1	PANI/RGO/PMFT	BC/PAM/H_2_SO_4_	1	564	-	-	-	[54]
2	MXene (Ti_3_C_2_Tx)/cellulose nanofiber/porous carbon film	PVA/KOH	0.1	143	-	0.3	72.1	[55]
3	Reduced graphene oxide-cellulose nanofibers	PVA/H_3_PO_4_	-	120	100	-	-	[56]
4	Twisting CNT@BC membrane/Ppy	PVA/H_2_SO_4_	0.8	458	-	-	-	[57]
5	Nonwoven cellulose/graphene/MnO_2_	PVA/H_2_SO_4_	0.5	139	-	-	-	[58]
6	Cellulose acetate/chitosan/rGO/NiO/Fe_3_O_4_	PVA/NaNO_2_	-	17	5	-	-	[59]
7	MnO_2_/Carbon fiber	PVA/H_2_SO_4_	-	-	-	1.5	20.5	[60]
8	CNT/CNF-MnO_2_ NWs	PVA/LiCl	0.5	619	-	-	158.7	This work

PMFT = poly(diallyldimethylammonium chloride)-modified fibre textile. BC= bacterial cellulose. PAM = polyacrylamide. Ppy = polypyrrole.

## Data Availability

Not applicable.
